# Unanticipated similarities and expected differences in the taxonomic composition and potential toxicity of cyanobacteria in biological soil crusts across hot and cold deserts

**DOI:** 10.3389/fmicb.2026.1766534

**Published:** 2026-03-11

**Authors:** Małgorzata Sandzewicz, Łukasz Łach, Nataliia Khomutovska, Małgorzata Suska-Malawska, Jan Kwiatowski, Hikmat Hisoriev, Iwona Jasser

**Affiliations:** 1 Faculty of Biology, Institute of Environmental Biology, University of Warsaw, Warsaw, Poland; 2 Department of Plant Protection Biology, Swedish University of Agricultural Sciences, Lomma, Sweden; 3 Department of Flora and Systematic Botany, Institute of Botany, Plant Physiology and Genetics, Tajikistan National Academy of Sciences, Dushanbe, Tajikistan

**Keywords:** 16S rRNA gene, biological soil crusts, cyanobacteria, cyanotoxins, diversity, soil properties, V3-V4 hypervariable region

## Abstract

The hot deserts of California and the cool, mountainous deserts of the Eastern Pamir region are geographically and climatically distinct, yet they share a common feature. Their arid soils host pioneering microorganisms that form biological soil crusts (BSCs), one of the earliest forms of life in this biome. This study aimed to reveal and compare the taxonomic composition, structure, and potential toxicity of cyanobacteria in these distant deserts, using simultaneous analysis. We observed significant differences in soil chemical properties, with higher average electrical conductivity in California and higher median levels of iron, nitrogen, carbon, magnesium, sodium, potassium, and calcium in Pamir. Despite this, the taxonomic composition and structure of the core bacteria phyla were similar, with Pseudomonadota, Actinomycetota, Bacteroidota, and Cyanobacteriota dominating in both locations. However, at the family level, bacterial communities showed more variability. Within Cyanobacteriota, the most abundant groups in California’s biocrust samples were unidentified families, followed by Nostocaceae, Coleofasciculaceae, Chroococcidiopsidaceae, and Phormidiaceae. In Pamir, Nodosilineaceae and Nostocaceae dominated, with a lower contribution from unknown families. In samples from both deserts, we identified cyanobacterial species known to produce cyanotoxins, along with the genes *mcy*E + *nda*F and *mcy*D, which are responsible for the microcystin and nodularin biosynthesis pathways.

## Graphical abstract

**Figure:**
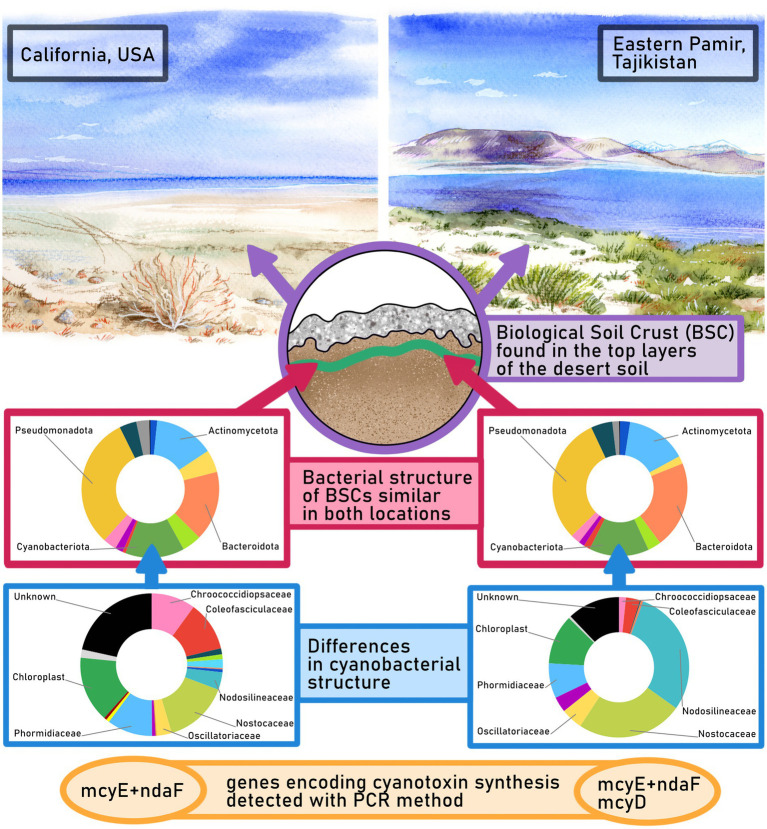

## 1 Introduction

Arid and semi-arid environments are the most common terrestrial habitats on Earth, covering about one-third of the planet’s land area. According to the Köppen climate classification (Köppen, 1931), arid climates are divided into deserts (BW) and steppe (BS), which can be either hot (h - with annual temperatures above 18 °C) or cold (k - with annual temperatures below 18 °C). An example of ecoregions with hot, arid climates includes the deserts in Southern California, United States—the Sonoran and Mojave. In June, the hottest month, average temperatures (from 1991 to 2020) ranged between 33 °C and 38 °C, with the lowest averages of 13 °C and 11 °C in January. During this period, average monthly rainfall varied from zero to no more than 16 mm in both regions (PRISM Climate Group, Oregon State University, 2024). These areas are among the hottest and driest in the world. Conversely, cold deserts and steppes are found in the Eastern Pamir Mountains of Tajikistan. This part of the Pamir Mountain range is a plateau above 3,500 meters elevation, surrounded by glaciers and mountain peaks covered in snow year-round (Normatov and Normatov, 2020). The average temperature here, based on data from the Murgab and Karakul meteorological stations, ranges from about −18 °C in January to 12 °C in June. In contrast, the average monthly rainfall remains below 20 mm throughout the year (Aichner et al., 2021), with temperatures at Bulunkul meteorological station dropping to −23.1 °C in January, and reaching an absolute minimum of −53 °C, while the highest average temperature in July is 10.0 °C, with an absolute maximum of 28.8 °C (Mętrak et al., 2023a).

Both the hot deserts of California and the cold deserts of Eastern Pamir Mountains experience extreme climatic conditions that foster the development of specialized microbial communities capable of surviving in such environments. Biological soil crusts (BSCs) are examples of these communities (Belnap and Lange, 2003; Mętrak et al., 2023b). They consist of organisms that inhabit the surface layers of soil and are adapted to harsh conditions like low moisture, prolonged soil desiccation, intense solar radiation, freeze–thaw cycles, or high salinity (Weber et al., 2022). Although BSCs are found worldwide, covering about 12% of the Earth’s land surface (Rodriguez-Caballero et al., 2018), their resilience in arid conditions has made them the oasis of life in desert biomes. BSCs are composed of autotrophic and heterotrophic bacteria, as well as green algae, lichens, mosses, liverworts, and microfungi. They play a vital role in desert ecosystems by stabilizing soil and enhancing its fertility (Belnap, 2003). The taxonomic structure of biological soil crusts can also be influenced by physical and chemical soil properties, as well as vegetation cover. In the hot deserts of California, biological soil crusts are usually flat and covered with pebbles due to the absence of soil freezing or rugose when lichens and mosses are present. The vegetation in this region is described as shrublands and mixed thorn scrubs (Belnap et al., 2001). The mountains of Eastern Pamir may be covered by desert vegetation, saline meadows, sedge meadows, and salt marshes. Soil in these desert areas is characterized by low organic carbon content and high electrical conductivity in surface layers (Mętrak et al., 2023a; Kabała et al., 2021).

Cyanobacterial soil crusts are primarily composed of cyanobacteria, classified as taxa within the Oxyphotobacteria class, along with heterotrophic Vampirivibrionia (formerly Melainabacteria) and Sericytochromatia. Oxyphotobacteria are gram-negative, photoautotrophic bacteria that can perform oxygenic photosynthesis. Cyanobacteria in BSCs often coexist with other bacterial phyla such as Acidobacteria and Pseudomonadota (previously Proteobacteria) (Powell et al., 2013). These bacteria contribute to decomposition, photosynthesis, and nitrogen fixation in anaerobic environments. For instance, filamentous *Microcoleus* sp. can produce thick extracellular sheaths composed of exopolysaccharides that bind and stabilize soil particles, boost nutrient availability, and enhance water absorption capacity, forming a cyanosphere that attracts other bacteria (Mager and Thomas, 2011; Büdel et al., 2016; Nelson and Garcia-Pichel, 2021). Another cyanobacterial group, *Nostoc* species, inhabits the upper crust layer and provides protection against excess ultraviolet radiation by producing special pigments (Belnap, 2003). Oxyphotobacteria can survive extended periods of desiccation and rapidly resume photosynthesis within minutes after water reenters (Abed et al., 2014). They also produce secondary metabolites, including various toxins like cyclic peptides (microcystins and nodularins) and alkaloids (anatoxins, cylindrospermopsin, saxitoxins, aplysiatoxins, lyngbyatoxins). Microcystins are mainly produced by *Microcystis aeruginosa*, *M. wesenbergii*, and *M. viridis* (van Apeldoorn et al., 2007). Other taxa with genes enabling toxin production include *Leptolyngbya* spp. (Magonono et al., 2018); *Nostoc* spp. (van Apeldoorn et al., 2007; Cirés et al., 2017; Chrapusta et al., 2015; Kaasalainen et al., 2013; Hisbergues et al., 2003); and *Phormidium* spp. (Chrapusta et al., 2015; Izaguirre et al., 2007). Nodularin production is associated with *Nodularia spumigena* and other *Nodularia* species (van Apeldoorn et al., 2007; Cirés et al., 2017; Kocasari et al., 2015), as well as *Nostoc* sp. (Gehringer et al., 2012). Among the most studied alkaloids, cyanobacteria frequently producing anatoxins include *Anabaena* spp., *Oscillatoria* spp., *Cylindrospermum* spp., *Aphanizomenon* spp., and *Phormidium* spp. (van Apeldoorn et al., 2007; Cirés et al., 2017; Chrapusta et al., 2015; Wood et al., 2012; Rantala-Ylinen et al., 2011). Cylindrospermopsin, another alkaloid, was first isolated from a strain of *Raphidiopsis* (formerly *Cylindrospermopsis*) *raciborskii*. This cyanobacterium is also known to produce saxitoxins alongside *Anabaena* spp. and *Aphanizomenon* spp. (van Apeldoorn et al., 2007; Kim et al., 2018).

Cyanotoxins are mainly studied in relation to algae blooms in water reservoirs. However, toxin-producing cyanobacteria and cyanotoxins can also be found in biocrusts on desert soils (Chatziefthimiou et al., 2021). The increase of cyanotoxin concentration in soil beneath biocrusts can be very slow, but over time, it can cause potential harm if the crust is disrupted and the dust particles containing toxins become airborne or enter water sources (Richer et al., 2014). In the deserts of Eastern Pamir Mountains, the primary water sources for local populations are rivers and lakes supplied by melting snow. Due to climate change, snow cover in the Pamir Mountains is decreasing (Normatov and Normatov, 2020). The combination of water scarcity and the presence of crust-forming cyanobacteria around water sources (Khomutovska et al., 2021) may pose a potential danger to humans and animals. Effects of climate change have also been observed in the Sonoran and Mojave Desert regions (Martinez-Austria and Bandala, 2017). Cyanobacteria in biocrusts are well adapted to extreme temperatures, and their toxin production can occur across a broad temperature range, even 10–35 °C in laboratory conditions (Cirés et al., 2017). However, it remains unclear which terrestrial species can produce these toxins or what factors trigger their production. Dulic et al. (2022) emphasized the need to examine the potential for cyanotoxin production in terrestrial environments, as we cannot assume that strains producing toxins in aquatic environments do so in biocrusts. For these reasons, we believe it is crucial to study biocrust cyanobacteria, their potential toxicity, and how it varies over time and under different environmental conditions.

We studied components of biological soil crusts: cyanobacteria and heterotrophic bacteria communities in two deserts—a cool, mountainous desert in Eastern Pamir and a hot desert in California. The study aimed to analyze and compare the diversity and structure of these communities in relation to soil chemical properties. We hypothesized that different environmental conditions would lead to differences in the composition and structure of bacterial communities in the BSC. The second goal was to assess the potential of the crusts’ cyanobacteria to produce cyanotoxins by identifying taxa previously known as toxin producers, as well as genes encoding toxin biosynthesis pathways. In our previous studies on microbial mats in Eastern Pamir, we successfully identified such taxa and genes, so we expected to find them in biological soil crusts as well. This study compares biocrusts from a well-studied region in North America with those from a less-explored area in Central Asia, linking the results to existing research and offering a broader understanding of biocrust-forming organisms worldwide.

## 2 Materials and methods

### 2.1 Sampling

Sampling was conducted in 2015 and 2017, with collections in California during January and March, and in the Pamir in July of both years. Soil crusts were collected at random from various locations within both desert types and placed into Petri dishes. They were dried in the open air, covered with dense nets to prevent contamination by insects or larger particles, and then sealed in plastic bags.

**Figure 1:**
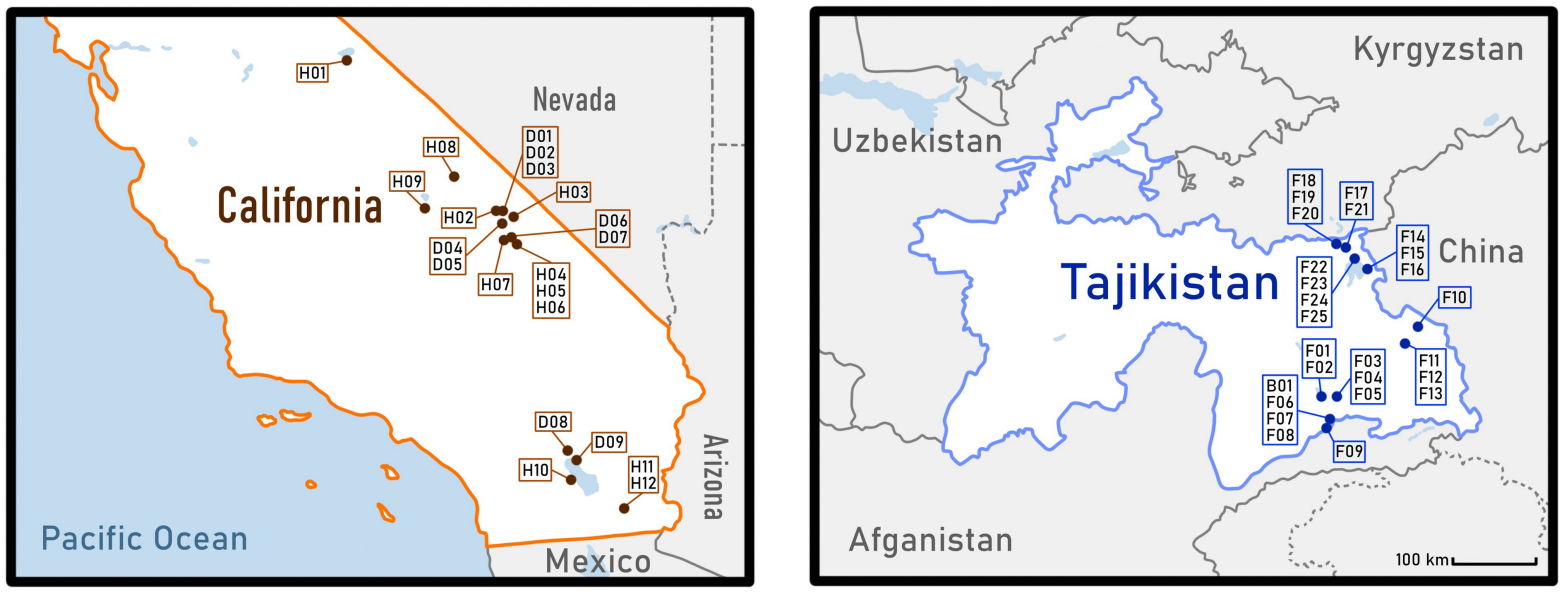
Maps of sample locations. On the left: the southern part of California State in the USA. On the right: the country of Tajikistan. Both maps have the same scale and were based on public domain pictures from WikiMedia (https://commons.wikimedia.org).

26 samples were collected from sites in Tajikistan and 21 samples in California, USA (Figure 1). The geographical location of each site was recorded using a GPS receiver [Garmin Trex 10 (010–00970-00)] as well as a multifunctional mobile navigation app, Locus Map (Supplementary Table S1). Most Californian sampling sites were in the Mojave ecoregion, including Death Valley and the area around Owens Lake. Five samples came from the region near the Salton Sea, which is part of the Sonoran Desert. According to the Köppen-Geiger Climate Classification, the sampling locations are characterized as arid climates—hot deserts (BWh) and hot steppes (BSh). The last site was in the Great Basin ecoregion near Mono Lake (Csb—warm temperate climate with dry and warm summers). Samples were collected in the Eastern Pamir Mountains of the Badakhshan Autonomous Region, Tajikistan, near the lakes Sassykkul, Bulunkul, Khargush, Chukurkul, Rangkul, Shorkul, and Karakul. These sites are described as cold deserts (BWk), cold steppes (BSk), and, for samples collected near Khargush Lake, polar tundra (ET).

For 24 biocrust samples from Tajikistan and 10 samples from California, corresponding soil samples were collected from underneath the biocrust to analyze the chemical parameters at each sampling site. Both types of samples - soil and BSC - after being shipped to the laboratory, were stored in a freezer prior to analysis.

### 2.2 Morphological analysis

The previously dried biocrust samples were revived by placing parts of them on Petri dishes with a solid WC medium (Guillard and Lorenzen, 1972). Once cyanobacterial colonies appeared, they were isolated and cultivated separately as described by Sandzewicz et al. (2023). Morphological analysis was conducted using an epifluorescence microscope Nikon ECLIPSE Ni-U 930941 (Tokyo, Japan). The identification of cultured cyanobacteria was based on the keys by Komárek and Anagnostidis (1998, 2005) and Komárek (2013).

### 2.3 DNA extraction, sequencing, and 16S rRNA gene amplicon analysis

The dried biocrust samples were used for DNA extraction using two kits: the E.Z.N.A.® Soil DNA Kit (Omega Bio-tek, Norcross, GA, USA) and the Soil DNA Purification Kit (GeneMATRIX, EURx Ltd., Gdańsk, Poland). Primers and DNA polymerase for amplifying the V3-V4 region of 16S rRNA were 341f and 785r primers (Klindworth et al., 2012) and HotStarTaq DNA Polymerase (Qiagen, Hilden, Germany). The process is detailed in our previous paper (Sandzewicz et al., 2023). The PCR product was sent to the Science and Technology Park “Bionanopark” (Łódź, Poland) for sequencing on the Illumina MiSeq platform (2 × 300), with a targeted sequencing depth of 150,000. Raw data are available in the BioProject database under the number PRJNA1304903. For bioinformatics analysis, paired-end reads were processed in the QIIME2 environment (ver. 2020.11). The data were then denoised and trimmed using DADA2 (Callahan et al., 2016). A SILVA-based classifier (ver. 138) for QIIME2 was used for taxonomic classification of the amplicon sequence variants (ASVs). Additionally, for genus- and species-level identification, cyanobacterial sequences were verified against the manually curated Cyanobacteriota reference package for phylogenetic analysis, Cydrasil (Roush et al., 2021). The project was shared on iTOL and the access details are in the supplement materials (Supplementary Table S1) Statistical analyses were performed on the rarefied data.

### 2.4 Detection of genes encoding the cyanotoxin biosynthesis pathways

The polymerase chain reaction (PCR) method was also used to detect the presence of cyanotoxin-encoding genes (*mcy*E+*nda*F, *mcy*D, *sxt*A, *cyr*L, *ana*C). For this reaction, we used FastGene® Optima HotStart ReadyMix (Nippon Genetics Co., Ltd., Tokyo, Japan) and the following primers: HEPf, HEPr (Jungblut and Neilan, 2006); *mcy*DF*, mcy*DR (Rantala, 2004); *sxt*Af, *sxt*Ar (Al-Tebrineh et al., 2011); *cyr*Lf*, cyr*Lr (Jiang et al., 2014); *ana*C-gen F, *ana*C-gen R (Rantala-Ylinen et al., 2011). The strains used as a positive control were as follow: *Microcystis aeruginosa* CPCC 299 for *mcy*E+*nda*F gene; *Microcystis aeruginosa* SAG14.85 for *mcy*D gene; *Aphanizomenon gracile* NIVA-CYA 655 for *sxt*A gene; *Aphanizomenon flos-aquae* NIVA-CYA 626 for *cyr*L gene; and *Phormidium favosum* AWQC-PHO021 for *ana*C gene. More details can be found in our previous paper (Khomutovska et al., 2020). The cycling conditions included an initial denaturation at 95 °C for 3 min, followed by 30 cycles of DNA denaturation at 95 °C for 15 s, primer attachment (for *mcy*E+*nda*F, *mcy*D, *sxt*A - 56,5 °C, for *cyr*L - 48 °C, for *ana*C - 58 °C; 30 s), and elongation at 72 °C for 1 min, ending with a final elongation step at 72 °C for 1 min.

### 2.5 Soil chemistry analysis

The chemical analysis was conducted by the Laboratory of Biogeochemistry and Environmental Conservation at the University of Warsaw, following the methodology described by Sulwiński et al. (2020). It used a CHNS NA2500 Thermoquest elemental analyzer (CE Instruments, UK) for total C and N, and a Speedwave 4 microwave mineralizer (Berghoff, Germany) for measuring total Fe, Mg, Na, K, and Ca after wet digestion in nitric/hydrofluoric acids (10:1, vol:vol).

### 2.6 Statistical analyses

Alpha diversity was calculated using R (version 4.0.0) and the vegan package in RStudio (version 1.2.5042). RStudio was also used to assess the statistical significance of specific results with the Kruskal-Wallis test (significance threshold: *p*-value < 0.05), as well as to generate a PCA plot (packages FactoMineR, factoextra) and box plots (ggplot2). The bar graphs and pie charts were created with Google Spreadsheets. All graphs were edited, combined, or enhanced (or fully drawn, in the case of Venn diagrams) in Autodesk SketchBook software. The illustrations in the graphical abstract were made with watercolors and colored pencils on paper, based on images taken during sampling, depicting Salton Sea in California and Sassykkul Lake in Eastern Pamir Mountains.

## 3 Results

The chemical parameters of soil samples varied significantly between the two regions (Supplementary Figure S1). The mean electrical conductivity was higher in California, with a range from 2,230 to 2,570 μS/cm. In Pamir, the highest EC value reached 5,220 μS/cm; however, the other samples ranged from 31 to 1,530 μS/cm. Soil pH was similar, with a median around 7.9 in both regions. Nevertheless, the median levels of iron, nitrogen, carbon, magnesium, sodium, potassium, and calcium were higher in Pamir. Except for iron content, the highest values and the overall greater interquartile range were observed in soils from the Pamir.

**Figure 2:**
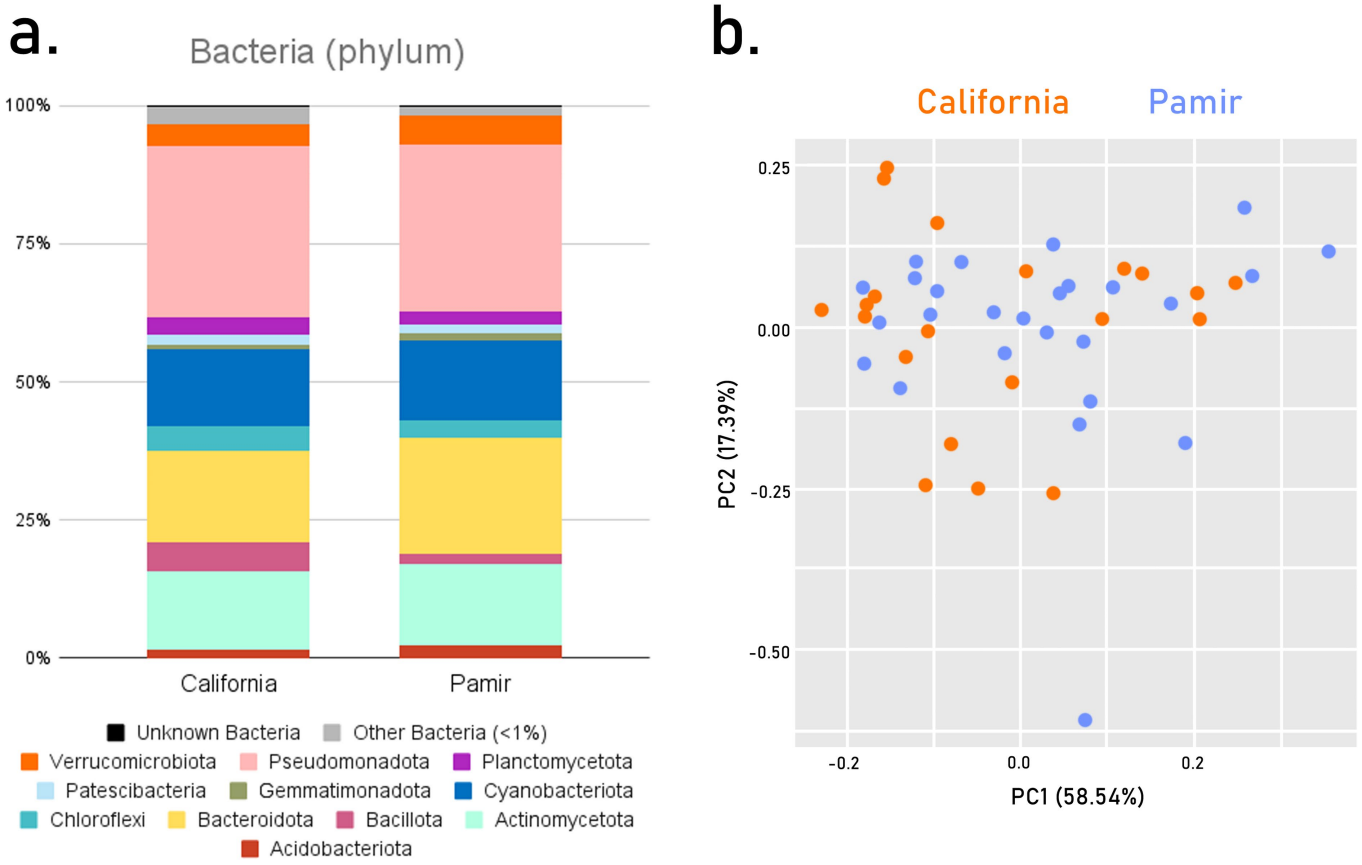
Taxonomic structure of BSCs at the phylum level for Bacteria, averaged across each environment **(a)**, and as a PCA for individual samples **(b)**.

The bacterial structure of soil crusts at the phylum level was similar in both environments (Figure 2a). The crusts were, on average, dominated by Pseudomonadota at around 30%, followed by Bacteroidota (21% in Pamir, 17% in California), Actinomycetota (formerly Actinobacteriota) (15% in Pamir, 14% in California), and Cyanobacteriota (14%). Among other bacterial phyla, Bacillota (foprevious Firmicutes) were more prevalent in California, while Gemmatimonadota were more common in Pamir. The unknown bacterial phyla made up 0.2% in each environment. The structure of individual samples at the phylum level was also very consistent across both environments (Figure 2b). There was no clear grouping that distinguished cold desert samples from hot desert samples. The four dominant phyla (Pseudomonadota, Bacteroidota, Actinomycetota, and Cyanobacteriota) contributed most to the sample distribution in the PCA.

**Figure 3:**
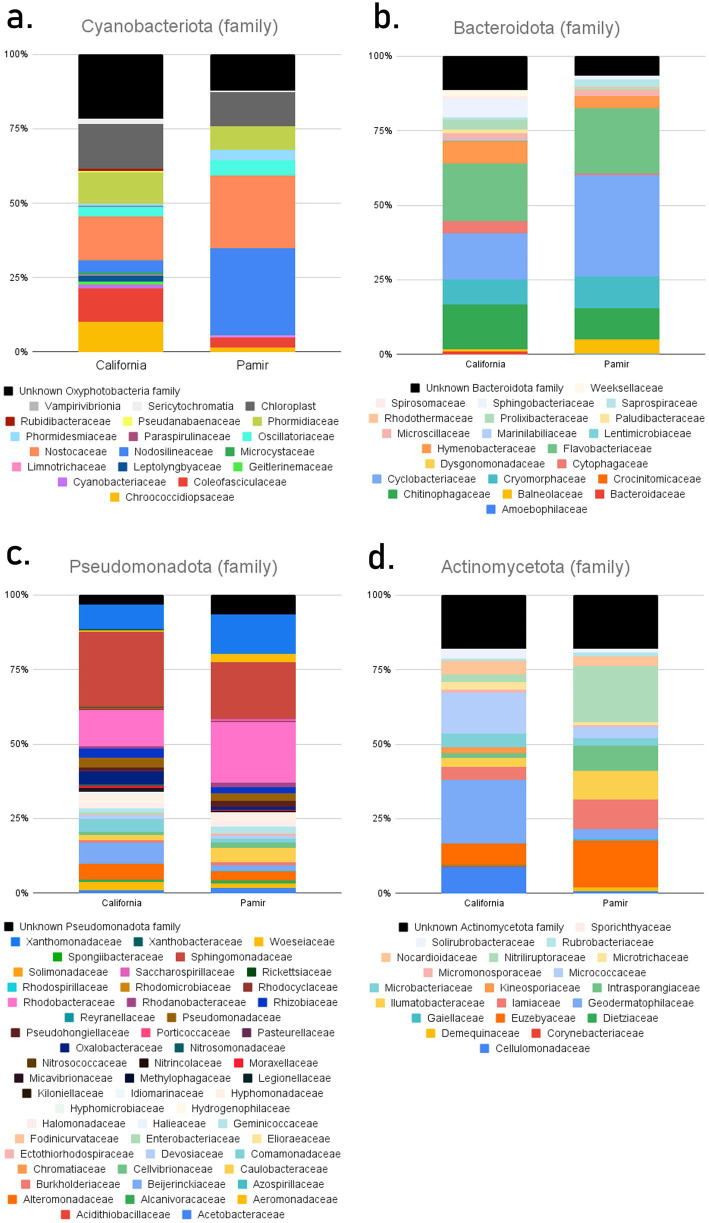
Family-level taxonomic structure of BSCs for the dominant phyla, averaged across each environment.

On the other hand, when focusing on one phylum—Cyanobacteriota—the structure of crusts from these two environments differed considerably (Figure 3a). In the hot desert crusts, 15 families of Oxyphotobacteria were identified, with Nostocaceae (14.7%), Coleofasciculaceae (11.3%), Phormidiaceae (10.2%), and Chroococcidiopsaceae (10%) being the most dominant. A large percentage (21.5%) of taxa were unknown Oxyphotobacteria families, along with chloroplasts (15%). Additionally, small proportions of Sericytochromatia (1.8%) and Vampirivibrionia (0.1%) were detected in these crusts. In cold desert environments, the percentage of Oxyphotobacteria taxa was higher, but the number of different families was lower — nine in total. Nodosilineaceae (29.2%) and Nostocaceae (24.4%) were the dominant families. The proportion of unknown Oxyphotobacteria was nearly half that of California, at 12.1%. There was also a lower percentage of chloroplasts (11.5%) and Sericytochromatia (0.5%).

Bacteroidota exhibited many structural similarities, differing mainly in the proportions of dominant families (Figure 3b). Flavobacteriaceae (19.2% in California, 21.8% in Pamir), Cyclobacteriaceae (15.6, 34%), and Chitinophagaceae (15, 10.4%) were dominant in both environments, with Cryomorphaceae (10.7%) also present in Pamir. Similar to Cyanobacteriota, a higher percentage of unknown families was found in California (11.4%) than in Pamir (6.5%).

Interestingly, Pseudomonadota had the highest number of identified families—52. However, only three were the most prevalent in both environments: Xanthomonadaceae (8.1, 13.4%), Sphingomonadaceae (25.1, 19.1%), and Rhodobacteraceae (12.2, 20.4%) (Figure 3c). This was also the only case where there was a higher percentage of unidentified families in Pamir (6.5%) than in California (3.4%).

The most notable difference between hot and cold climates was observed in Actinomycetota (Figure 3d). There was no overlap in dominant taxa. In California, the main families were Micrococcaceae (13.7%), Geodermatophilaceae (21.3%), and Cellulomonadaceae (8.9%). In Pamir, the dominant families were Nitriliruptoraceae (18.9%), Intrasporangiaceae (8.3%), Ilumatobacteraceae (9.7%), Iamiaceae (9.9%), and Euzebyaceae (15.7%). Both environments had the same percentage of unknown families, at 17.9%.

**Figure 4:**
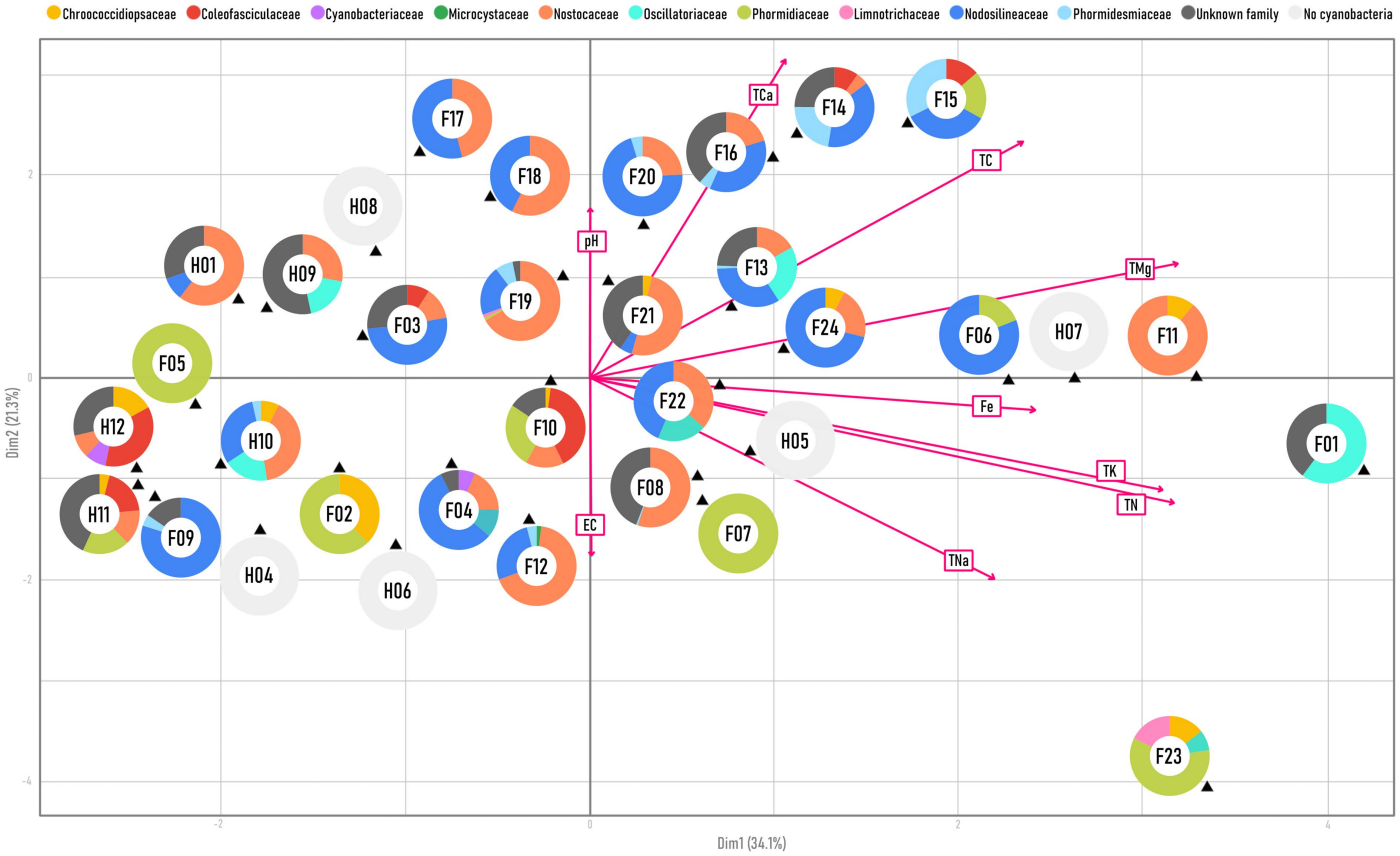
PCA of soil parameters (pH, total calcium, total carbon, total magnesium, iron, total potassium, total nitrogen, total sodium, electrical conductivity) of samples from Pamir (F__) and California (H__), with pie charts showing cyanobacterial structures at the family level of biocrusts corresponding to each soil sample.

The chemical parameter values for 24 soil samples from Pamir and 10 from California were used to perform a PCA, which revealed their similarities and was compared to the cyanobacterial structure of biocrusts collected from the same sampling sites (Figure 4). The placement of the samples along the y-axis was determined by pH and EC values. In contrast, the other parameters influenced their position along the x-axis. The samples did not form distinct clusters, and there was no clear separation between those from the Pamir and those from California. Although most of the California samples were located on the left side of the x-axis, the Pamir samples were more evenly distributed across the x-axis, covering a wider range of environmental parameters.

As a result, samples H11 and H12 from the Salton Sea showed closer similarity to the Pamirian sample F09 collected near Chukurkul Lake than the other sample from the Salton Sea, H10. This difference in soil parameters among individual samples was also evident within the same geographic regions. Samples from the Death Valley desert (H04, H05, H06, H07, H08) were scattered across the entire graph, remaining separate from each other, just like samples collected from the mountainside near Shorkul Lake (F11, F12, F13). Additionally, no pattern was observed in the placement of individual samples with similar taxonomic structures.

**Figure 5:**
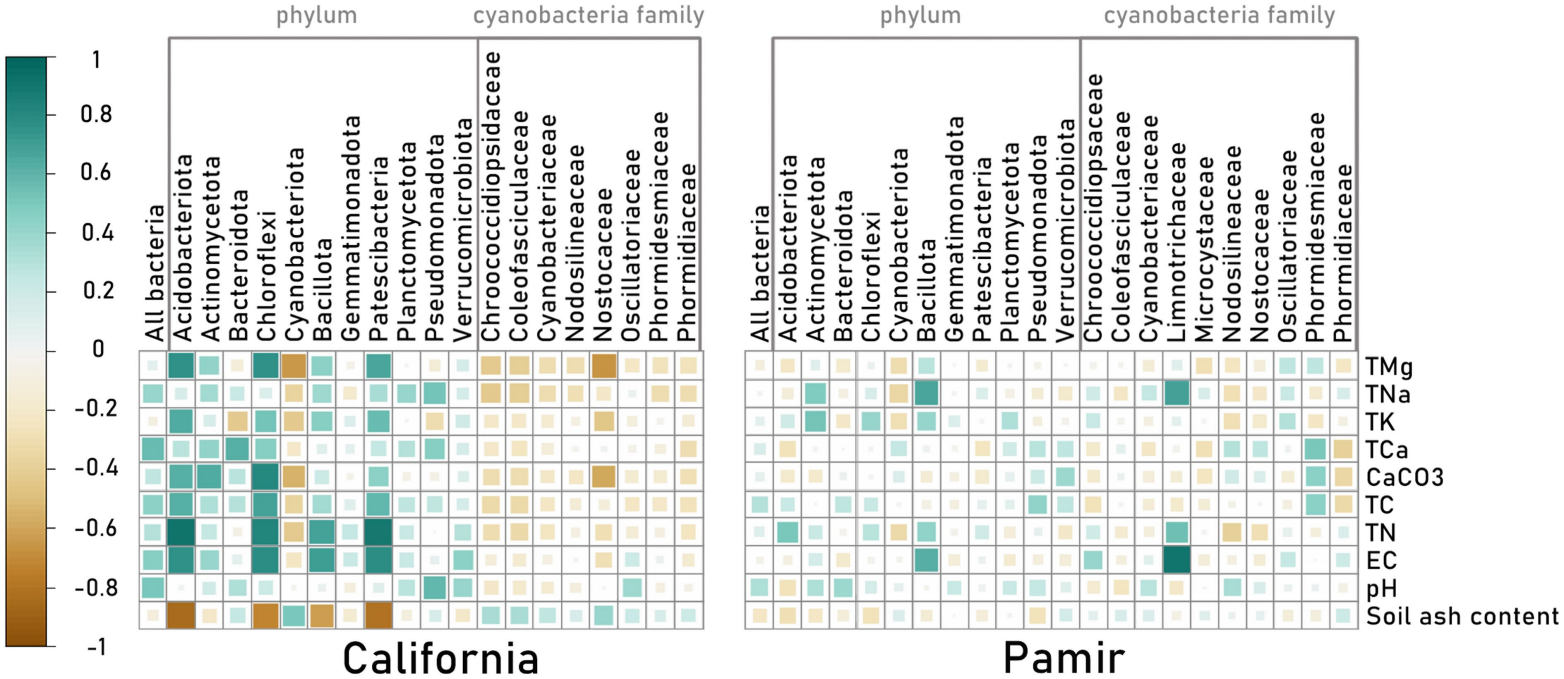
Correlation between combined taxonomic unit read counts and soil chemical properties for each geographic location.

On the other hand, combined values across different taxonomic units correlated more strongly with soil properties in California than in the Pamir (Figure 5). There was a significant positive correlation between the phyla Patescibacteria, Bacillota, Chloroflexi, and Acidobacteriota and TMg, electrical conductivity, and TN. The same phyla correlated negatively with soil ash content. Cyanobacteriota, however, showed the opposite relationship with soil chemistry, as reflected in one of the dominant families, Nostocaceae. The total number of bacterial reads from California also had strong positive correlations with TNa, TCa, TC, EC, and pH. In Pamir, the only strong positive correlations were between the family Limnotrichaceae of Cyanobacteriota and the phylum Bacillota, and TNa, EC, and TN.

**Figure 6:**
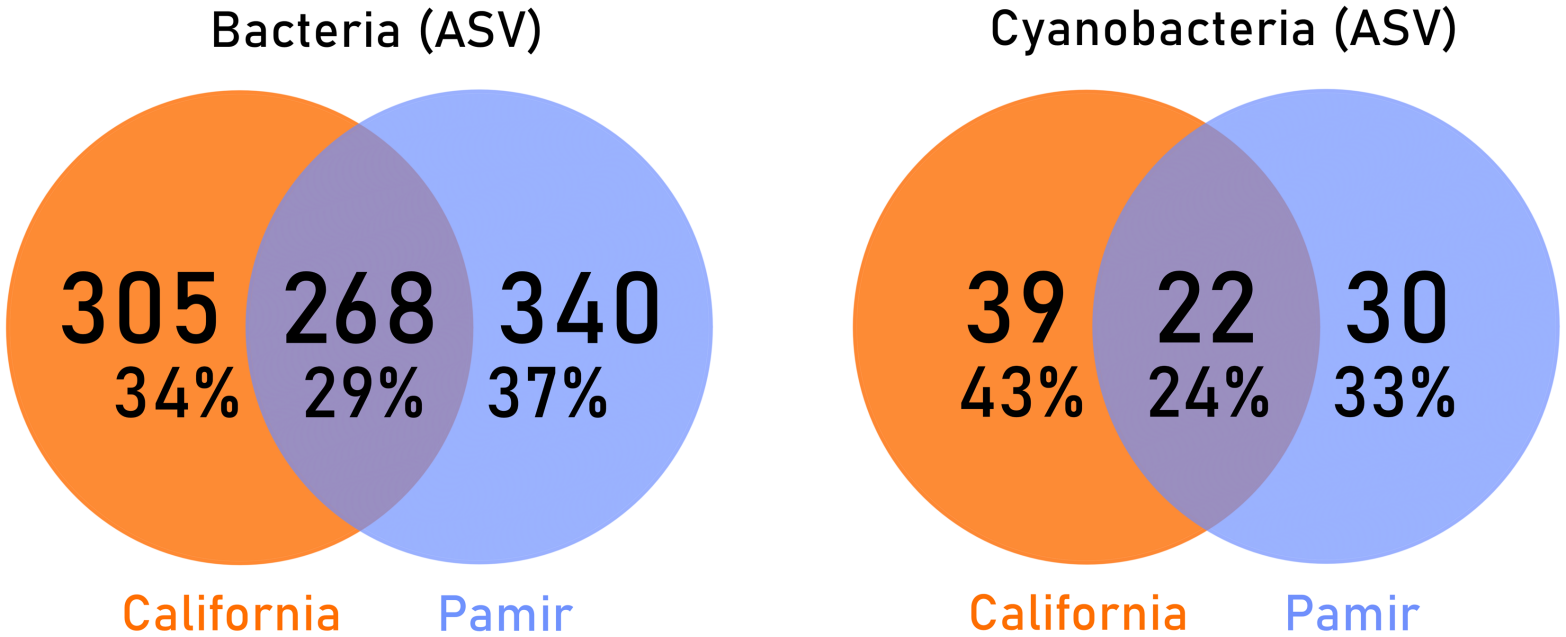
Amplicon Sequence Variants unique to each environment and shared between them - on the left: all bacteria including cyanobacteria; on the right: only cyanobacteria.

From 47 samples collected in California and the Pamir, a total of 913 distinct Bacterial ASVs were amplified, including 91 identified as Cyanobacteriota. The Venn diagrams in Figure 6 show the number and percentage of ASVs found exclusively in one environment and those shared between the two environments. Among all bacteria, the highest percentage of distinct ASVs (37%) was observed only in Pamir samples, while one-third of the taxa were unique to California deserts. The overlap accounted for the remaining 29%. When focusing specifically on cyanobacterial ASVs, less than a quarter of the taxa were shared between the two environments. The highest percentage of cyanobacterial ASVs was unique to California (43%), with 33% unique to Pamir (Figure 6).

The genes encoding the microcystin and nodularin biosynthetic pathways—*mcy*E and *nda*F—were detected in 13 samples, seven from Pamir and six from California (Table 1). Additionally, in five of the Pamirian biocrust samples, *mcy*D genes were amplified. In nearly all the mentioned samples, microscopic analysis showed the presence of potentially toxic cyanobacteria, mostly *Leptolyngbya* and *Phormidium* spp., but also *Nostoc* sp. The exception was sample F16, from which no cyanobacterial isolates were obtained. However, molecular methods did not detect potentially toxic cyanobacteria in six samples, despite their presence in cultures. In the remaining samples, sequences highly similar to various species of *Leptolyngbya* and *Nodularia spumigena,* were present.

**Table 1:** Potentially toxic cyanobacteria in samples in which genes encoding toxin biosynthesis were detected.

Nr	Genes detected (PCR)	Potentially toxic ASVs (possible MCs and NODs synthesis) - species similar to:	Potentially toxic genera detected in cultures
F06	*mcy*E+*nda*F	-	*Leptolyngbya* sp. *Phormidium* sp.
F07	*mcy*E+*nda*F, *mcy*D	-	*Leptolyngbya* sp.
F08	*mcy*E+*nda*F, *mcy*D	*Leptolyngbya* sp. CYN68 *Leptolyngbya tenuis* PMC304.07	*Leptolyngbya* sp. *Phormidium* sp.
F10	*mcy*E+*nda*F, *mcy*D	*Leptolyngbya tenuis* PMC304.07	*Leptolyngbya* sp.
F15	*mcy*E+*nda*F, *mcy*D	*-*	*Leptolyngbya* sp. *Phormidium* sp. *Nostoc* sp.
F16	*mcy*E+*nda*F, *mcy*D	*Leptolyngbya* sp. UIC 10125 *Leptolyngbya* sp. PCC 6406	NA
F18	*mcy*E+*nda*F	*Leptolyngbya* sp. UIC 10125	*Leptolyngbya* sp.
D01	*mcy*E+*nda*F	-	*Nostoc* sp.
D07	*mcy*E+*nda*F	*Leptolyngbya* sp.	*Leptolyngbya* sp.
D08	*mcy*E+*nda*F	-	*Leptolyngbya* sp.
D09	*mcy*E+*nda*F	-	*-*
H01	*mcy*E+*nda*F	*Nodularia spumigena*	*Nostoc* sp. *Phormidium* sp.
H12	*mcy*E+*nda*F	-	*Phormidium* sp.

Samples from Pamir: F__; samples from California: D__; and H__.

## 4 Discussion

The composition of cyanobacterial taxa in biological soil crusts tends to be quite similar across continents, as they are a very ancient group of organisms and may have functioned in these environments even before the division of Pangaea (Bowker et al., 2016). Moreover, their active growth is usually driven by similar temperature and moisture conditions, which can occur during different seasons in very different ecoregions (Belnap et al., 2001). The bacterial composition of biocrusts in cold environments like Antarctica generally includes Actinomycetota, Pseudomonadota, Bacteroidota, Acidobacteriota, Gemmatimonadota, Deinococcus-Thermus, and Cyanobacteriota (Bottos et al., 2014), with Cyanobacteriota making up to 20% of the crust’s prokaryotic reads (Jung et al., 2017). In Hulun Buir Sandy Land (China), Cyanobacteriota accounted for 23.8% of sequences, and Actinomycetota were dominant, followed by Pseudomonadota, Chloroflexi, Bacteroidota, and Acidobacteriota (Duan et al., 2024). The most abundant genera of Cyanobacteriota (Microcoleus_PCC-7113, norank_Coleofasciculaceae, Crinalium_SAG_22.89), Actinomycetota (norank_Frankiales, norank_Vicinamibacterales, unclassified_Micromonosporaceae), and Pseudomonadota (norank_Acetobacteraceae, Acidiphilium, Microvirga, and Candidatus_Alysiosphaera) in this cold desert were also identified using the SILVA classifier in our samples from the Eastern Pamir Mountains.

In hot environments, Cyanobacteriota can also be a dominant phylum, usually followed by a significant percentage of Pseudomonadota, Actinomycetota, and Chloroflexi, as seen in the Sonoran Desert (Nagy et al., 2005), Colorado Plateau (Gundlapally and Garcia-Pichel, 2006), or the semi-arid region of the municipality of Irauçuba, Brasil (Pinheiro et al., 2024). In other biocrusts, such as those from the Northern Cape Province (South Africa), Bacteroidota, Pseudomonadota, and Actinomycetota were the dominant phyla, and Cyanobacteriota contributed, on average, less than 10% (Maier et al., 2018).

Our study revealed that the average bacterial composition of biocrusts at the phylum level across two deserts separated by thousands of kilometers was very similar, with core bacterial phyla Pseudomonadota, Bacteroidota, Actinomycetota, and Cyanobacteriota resembling those of the Northern Cape Province. The results suggest that a conserved core at the phylum level of desert biocrust bacteria may exist despite climatic differences, as evidenced by the simultaneous analysis of samples from these two regions. A similar core comprising of: Actinomycetota (Actinobacteriota), Pseudomonadota (Proteobacteria), Bacteroidota were observed in BSC in Sonora Desert in Mexico (Ramos-Madrigal et al., 2024) and in cold desert Taklamakan Desert (Zhang et al., 2025). Also other authors studying phyllosphere communities observed similar core bacterial communities in seven ecosystems (Xue et al., 2023). The differences between California and Pamir within these core phyla were notable at the family level (Figure 2a). The main differences involved a more diverse structure, higher taxonomic richness, and a more even distribution in the hot desert compared to the cold desert. In the cold desert, over half of the taxa belong to a few dominant families, except for Actinomycetota, where the distribution of taxa is more uniform in Pamir than in California.

The most common cyanobacteria in biocrusts are *Microcoleus vaginatus* and *Nostoc* spp. (Belnap, 2003; Büdel et al., 2016), which are believed to be key components during their early formation. For example, *M. vaginatus* was identified as a prominent cyanobacterium in biocrusts on the Colorado Plateau in the USA (Gundlapally and Garcia-Pichel, 2006), Bafq Region in Central Iran (Moghtaderi et al., 2009), Gurbantunggut Desert, China (Zhang et al., 2006, 2010), and the Tibetan Plateau (Čapková et al., 2016). Other species commonly found worldwide belong to genera *such as Coleofasciculus*, *Leptolyngbya, Phormidium*, *Chroococcidiopsis,* and *Schizothrix* (Büdel et al., 2009; Xu et al., 2021; Mugnai et al., 2018; Hagemann et al., 2015; Pushkareva et al., 2015; Patova et al., 2023; Rippin et al., 2018). In our samples from the Pamir, the highest number of identified reads belonged to *Microcoleus acremanii* UTCC 313 (Supplementary Table S1). Other prevalent cyanobacteria included *Nodularia spumigena* and various *Leptolyngbya* species. The latter were commonly found in samples from California, along with *Chroococcidiopsis* sp., *M. acremanii* UTCC 313, *N. spumigena,* and *Wilmottia* sp. CAWBG522. Through microscopy, we observed, isolated, and cultured various cyanobacteria from *the Nostoc* and *Phormidium* genera, even though they did not appear in the molecular analysis results. These species were common in our cultures from both environments, alongside *Leptolyngbya*. Additionally, we detected genera such as *Scytonema* and *Calothrix* in both desert types. A similar discrepancy between molecular and microscopic findings was noted in our previous studies in Pamir, particularly in microbial mats (Jasser et al., 2020; Sandzewicz et al., 2023). As discussed then, this may result from a lack of benthic cyanobacteria sequences in databases and difficulties in isolating DNA from taxa with very thick gelatinous sheaths, which are characteristic of benthic and biocrust cyanobacteria. Identifying biocrust taxa can also be hampered by the low number of cells many species produce, making standard identification techniques insufficient. Furthermore, less research interest in soil organisms—compared to aquatic ones—has led to fewer data being available in databases for comparison (Belnap and Lange, 2003).

Since the formation of modern continents, desert cyanobacteria have independently adapted to local climatic conditions while being subjected to environmental selection, leading to differences in taxonomic composition. Bahl et al. (2011) examined the ancestry of Chroococcidiopsis strains from different continents and discovered that variants from hot and cold deserts were evolutionarily distinct. This might explain the trend in our results, which show high similarity among bacterial communities at higher taxonomic levels. At the phylum level, the average bacterial composition of biocrusts from California and Pamir showed few differences (Figure 2a). However, at the lowest level (ASVs), only 29% of bacterial species were found in both hot and cold deserts (Figure 6). Even fewer species (24%) were shared between the two environments when focusing solely on cyanobacteria. The differences become more evident at the family level, where in Pamirian biocrusts, most cyanobacteria belonged to Nodosilineaceae and Nostocaceae, whereas in California, dominant families included Nostocaceae, Coleofasciculaceae, Phormidiaceae, and Chroococcidiopsaceae (Figure 3a). Notably, these families often contain diazotrophic taxa. In California, Nostocaceae and Chroococcidiopsidaceae together accounted for approximately 24.7% of all cyanobacterial ASVs. In contrast, in Pamir, Nostocaceae alone accounted for 24.4%, and the phylum was co-dominated by Nodosilineaceae (29%), which includes taxa with nitrogenase. Therefore, the cyanobacteria community in Pamir may have a greater potential for atmospheric nitrogen fixation (Perkerson III et al., 2011). This is interesting, as the soil analysis revealed a higher median TN in the Pamir than in California, and we expected a higher potential for N_2_ fixation in an environment with more limiting nitrogen resources. However, while the prevalence of Nostocales in BSCs in Pamir and their contribution to N_2_ fixation is not disputed, the nitrogen-fixing capacity of other taxa across these families remains and their contribution is uncertain without analysis of nitrogenase genes. Therefore we can not preclude that the apparent higher potential of cyanobacteria in the Pamir to fix nitrogen is overestimated.

The separation of biomes and the specialization of biocrust cyanobacteria could also be related to the high percentage of unidentified taxa (Garcia-Pichel et al., 2003). In California and Pamir, 21. 5% and 12.1. 1% of cyanobacteria, respectively, could not be matched to any families in the database. Interestingly, in California, nearly twice as many cyanobacterial sequences were unidentified at the family level compared to Pamir. This is surprising, given that the cold deserts of Tajikistan are much less studied than the hot deserts of North America, with fewer sequences in public databases. We expected more new, unidentified taxa in the remote Pamir Mountains. The greater diversity of Cyanobacteria in California compared to Eastern Pamir is also notable, because in desert ecosystems, BSC depend on water availability during the vegetation season and temperature (Barrón-Sandoval et al., 2023). The seemingly milder environmental conditions in the Pamir Mountains, with smaller daily temperature amplitudes and higher average annual precipitation than in California, do not appear to result in greater microbial taxonomic diversity. This may be related to the annual rainfall/precipitation distribution and actual water availability during the vegetation season. The precipitation intensity in both studied deserts occurs mainly in winter months (Warner, 2009), but while the vegetation season in the hot desert falls in winter/spring, in the cold desert it falls in summer. Thus, water availability during the vegetation season may explain differences in cyanobacteria diversity. Another interesting aspect of the diversity of organisms in desert ecosystems was recently presented by Gross et al. (2024), who found that the phenotypic and functional diversity of plants in arid environments was higher than in more mesic ecosystems. The authors suggested that extreme conditions and lower competition among organisms, associated with lower biomass, may lead to higher and unexpected phenotypic and functional diversity. This hypothesis is worth verifying in the case of microorganisms.

The strength of this study is that we use the same sampling techniques and a simultaneous analysis with the same molecular method to examine microbial communities from two distant locations. Because of that, we could confidently compare the cyanobacterial composition (family level) of each sample, from all locations, with chemical parameters of the soil (Figure 4). As it turned out, there was no visible link between similarly composed samples and a specific soil chemistry. It may be a clue that other, untested parameters could be influencing the dominance of certain families. Additionally, cyanobacteria such as Nodosilineaceae and Nosocaceae showed high ecophysiological plasticity occurring across a wide range of conditions. On the other hand, when looking at the two desert types separately, and collectively at different bacteria phyla and cyanobacteria families, some correlations with soil chemistry can be observed, especially in California (Figure 5). Interestingly in one of our previous studies on different BSC samples from similar locations in Eastern Pamir Mountains (Khomutovska et al., 2021) total carbon concentration correlated positively with bacterial reads. In our samples, total carbon explained only 32% of the combined reads for all bacteria, though, together with pH, it showed the highest correlation observed, which is partially in line with previous studies. There were also no other significant correlations with carbon, and all R-squared values ranged from −0.34 to 0.46. Summarizing, the hot desert was characterized by stronger correlations between taxon reads at various levels and soil parameters. In contrast, in the cold desert, the relationship between taxonomic composition and soil properties seemed less pronounced.

Our second aim in the study was to assess the potential toxicity of cyanobacteria in biological soil crusts. Detecting cyanotoxins in desert soil presents many challenges. Water availability in arid and semi-arid areas can significantly influence the ability of crust microorganisms to produce toxins (Dulic et al., 2022). Cyanotoxins and genes related to their synthesis have been found in biocrusts from both hot and cold environments, such as microcystins and *mcy*D in the Qatar Desert (Metcalf et al., 2012), and MC-LR and ANTX-a in the Kaffiøyra Plain, NW Spitsbergen (Chrapusta et al., 2015). Microcystin-encoding genes also appeared in our samples from both environments (Table 1). The detection of *mcy*E + *nda*F and *mcy*D genes in Pamir was expected, as these had been identified in our previous study of microbial mats collected during the same field trip (Khomutovska et al., 2020). However, that paper and another from this region implied that high altitude might not only limit toxin production but also the occurrence of genes involved in toxin biosynthesis pathways (Khomutovska et al., 2020; Jasser et al., 2024), as in the stressful environment, the production of costly secondary metabolites such as toxins might be useful. Nonetheless, the hepatotoxin-encoding genes in the studied BSCs were amplified in about 30% of samples from both environments, suggesting that both the hot- and cold-desert cyanobacteria experience extreme conditions leading to lower occurrence of toxic genotypes. No neurotoxin-encoding genes were detected in BSCs. Quantifying cyanotoxins in soil and BSCs can be difficult, as no single method has been proven to work across different soil types (Zhang et al., 2020). Consequently, further research and the development of reliable testing methods are needed. Until then, the presence of potentially toxic taxa cannot be overlooked. While we did not detect Microcystis, the most common microcystin-producing cyanobacteria in aquatic environments, the BSC samples from Pamir and California showed a high abundance of Leptolyngbya, Phormidium, and Nostoc species (Table 1), all previously shown to produce MCs. This highlights the difficulty of drawing conclusions about terrestrial cyanobacteria, especially given the scarcity of comparable data and the fact that most information is based on toxic aquatic cyanobacteria. Therefore, expanding our understanding of the potential toxicity of biocrust cyanobacteria is essential, particularly given climate change and the widespread desertification affecting many terrestrial ecosystems.

## 5 Conclusion

In conclusion, by analyzing and comparing the structures of biological soil crust communities from hot and cold deserts in relation to environmental factors, we found a common bacterial core community at the phylum level in both regions; however, differences emerged at lower taxonomic ranks. Despite differences in cyanobacterial composition at the family level, no clear pattern emerged that would indicate that the same soil chemical properties influenced groups of samples resulting in similar cyanobacterial communities. Overall, the relationship between taxonomic units from samples of the same desert type and the specific soil properties was more apparent in California than in the Pamir. The higher diversity of cyanobacteria in the hot desert than in the cold desert might be linked to the fact that the vegetation season in the southwestern deserts of the United States falls in winter/spring, when rainfall is possible. In contrast, in the Pamir desert, it occurs during the short summer, when rainfall is absent.

We also assessed the potential of BSC cyanobacteria to produce cyanotoxins by identifying taxa known to be toxin-producing as well as genes involved in toxin synthesis. We amplified the *mcy*E+*nda*F gene in both desert types and the *mcy*D gene in samples from the Eastern Pamir Mountains. In samples from both regions, we found several ASVs with high similarity to species known to produce microcystins or nodularins. Additionally, we isolated potentially toxic genera from environmental samples.

However, there is currently insufficient information on toxin-producing cyanobacteria growing in desert biocrusts to confidently assess the risk of cyanotoxins to humans and animals. Our findings indicate only the potential for toxin biosynthesis and should therefore be regarded as the basis for a hypothesis that could be tested in future studies. Consequently, continuing research in this area is crucial, especially in the context of climate variability and global environmental changes.

## Data Availability

The datasets presented in this study can be found in online repositories. The names of the repository/repositories and accession number(s) can be found in the article/Supplementary material.
