# Supplementary material for: Unanticipated similarities and expected differences in the taxonomic composition and potential toxicity of cyanobacteria in biological soil crusts across hot and cold deserts
Source: Front Microbiol. 2026 Mar 11;17:1766534. doi: 10.3389/fmicb.2026.1766534 (PMC13013331; doi:10.3389/fmicb.2026.1766534)

Unanticipated similarities and expected differences in the taxonomic composition and potential toxicity of cyanobacteria in biological soil crusts across hot and cold deserts.

Małgorzata Sandzewicz^1*^, Łukasz Łach^1^, Nataliia Khomutovska^1,2^, Małgorzata Suska-Malawska^1^, Jan Kwiatowski^1^, Hikmat Hisoriev^3^ Iwona Jasser^1^

^1^Institute of Environmental Biology, Faculty of Biology, University of Warsaw, Warsaw, Poland

^2^Department of Plant Protection Biology, Swedish University of Agricultural Sciences, 23422 Lomma, Sweden

^3^Flora and Systematic Botany Department Institute of Botany, Plant Physiology and Genetics, Tajikistan National Academy of Sciences, Dushanbe, Tajikistan

* corresponding author: [m.sandzewicz@uw.edu.pl](mailto:m.sandzewicz@uw.edu.pl)

**Supplementary materials**

Tab.S1. Description of location of samples, climate characteristics, as well as Oxyphotobacteria genera and species identified morphologically (from cultures) and molecularly, by placing the sequences on a phylogenetic tree (Cydrasil) with a like-weight ratio > 95%.

| Sample | Year of collection | Location description | Latitude,  Longitude  (DD) | Oxyphotobacteria identified morphologically | Cydrasil matches |
| --- | --- | --- | --- | --- | --- |
| D01 | 2015 | Trail Canyon, Death Valley | 36.317175,  -116.921456 | *Scytonema* sp., *Trichormus* sp., *Nostoc* sp. | *Chroococcidiopsis* sp.,  *Microcoleus acremanii* UTCC 313 |
| D02 | 2015 | Death Valley | 36.272143,  -116.883917 | *Anabaena* sp., *Nostoc* sp. | *Synechococcus* sp. PCC 7336, *Pycnacronema arboriculum* 41PC, *Aerosakkonema funiforme* Lao35, *Leptolyngbya* sp., *L.* sp. CYN68, *Wilmottia* sp. CAWBG522 |
| D03 | 2015 | Death Valley | 36.282725,  -116.887284 | no observed Oxyphotobacteria | *Synechococcus* sp. PCC 7336, *Pycnacronema arboriculum* 41PC, *Nodularia spumigena*, *Microcoleus acremanii* UTCC 313, *Leptolyngbya* sp., *L.* sp. CYN68, *L.* sp. PCC 6406, *Wilmottia* sp. CAWBG522 |
| D04 | 2015 | Wash, Trail Canyon, Death Valley | 36.168137,  -116.880158 | *Microcoleus* sp., *Leptolyngbya* sp., *Nostoc* sp., *Nostoc edaphicum* | *Synechocystis* sp. PCC 7509, *Chroococcidiopsis* sp., *Parifilum* *solicrustae* SON57, *Pycnacronema arboriculum* 41PC, *Leptolyngbya* sp., *L.* sp. SAG 2411 |
| D05 | 2015 | Wash, Trail Canyon, Death Valley | 36.168137,  -116.880158 | *Nostoc* sp., *Chlorogloeopsis* sp., *Leptolyngbya* sp. | *Synechococcus* sp. PCC 7336, *Synechocystis* sp. PCC 7509, *Parifilum* *solicrustae* SON57 |
| D06 | 2015 | Death Valley | 36.006125,  -116.783142 | *Nostoc linckia*, *Isocystis* sp. | *Geitlerinema* sp., *Calothrix* sp. UAM 374, *Nodularia spumigena*, *Aerosakkonema funiforme* Lao35, *Microcoleus acremanii* UTCC 313, *Wilmottia* sp. CAWBG522, *Leptolyngbya* sp. SAG 2411 |
| D07 | 2015 | Death Valley | 35.964422,  -116.744562 | *Leptolyngbya* sp. | *Synechococcus* sp. PCC 7336, *Aerosakkonema funiforme* Lao35, *Microcoleus acremanii* UTCC 313, *Leptolyngbya* sp., *Wilmottia* sp. CAWBG522, *Pseudanabaena* sp. PCC 6802 |
| D08 | 2015 | Mecca Aqueduct | 33.620382,  -116.001011 | *Leptolyngbya* sp. | *Microcoleus acremanii* UTCC 313, *Wilmottia* sp. CAWBG522 |
| D09 | 2015 | the area around Salton Sea | 33.485244,  -115.878801 | *Calothrix* sp. | no matches with like-weight ratio above 95% |
| H01 | 2017 | the area around Mono Lake | 37.942215,  -119.0229 | *Nostoc punctiforme*, *Phormidium* sp. | *Synechococcus* sp. PCC 7336, *Nodularia spumigena* |
| H02 | 2017 | Trail Canyon Road, Death Valley | 36.312672,  -116.978516 | *Leptolyngbya* sp., unidentified Oscillatoriales | no matches with like-weight ratio above 95% |
| H03 | 2017 | Bad Water, Death Valley | 36.231552,  -116.766647 | *Nostoc punctiforme* | no matches with like-weight ratio above 95% |
| H04 | 2017 | Ashford Mill, Death Valley | 35.925684,  -116.676140 | *Pseudanabaena* sp. | no matches with like-weight ratio above 95% |
| H05 | 2017 | West Side Road, Death Valley | 35.936287,  -116.716873 | no observed Oxyphotobacteria | no cyanobacteria identified |
| H06 | 2017 | West Side Road, Death Valley | 35.941525,  -116.733124 | *Phormidium* sp. | no cyanobacteria identified |
| H07 | 2017 | Death Valley | 35.96489334,  -116.9087600 | no observed Oxyphotobacteria | no cyanobacteria identified |
| H08 | 2017 | Racetrack Playa | 36.673565,  -117.570358 | *Phormidium* sp. | no cyanobacteria identified |
| H09 | 2017 | the area around Owens Lake | 36.330063,  -117.949409 | *Phormidium* sp. | *Nodularia spumigena*, *Capilliphycus* sp., *Leptolyngbya* sp. CYN68, *L. tenuis* PMC304.07, *Loriellopsis cavernicola* LF-B5-WARN |
| H10 | 2017 | the area around Salton Sea | 33.289951,  -115.964897 | *Pseudanabaena* sp., *Microcoleus* sp. | *Rivularia* sp. VP4 08, *Leptolyngbya* sp. UIC 10125, *L.* sp. CYN68, *Loriellopsis cavernicola* LF-B5-WARN |
| H11 | 2017 | Salton Sea, Dunes, Holtville | 32.969704,  -115.264603 | unidentified Oscillatoriales | *Leptolyngbya* sp. SAG 2411, *Calothrix* sp. 96/26 LPP3 |
| H12 | 2017 | Salton Sea, Dunes, Holtville | 32.969704,  -115.264603 | *Phormidium* sp. | *Chroococcidiopsis* sp. |
| B01 | 2015 | the area around Sassykkul Lake | 37.40685,  73.10833 | *Leptolyngbya* sp. | *Nodularia spumigena*, *Leptolyngbya* sp. UIC 10125 |
| F01 | 2017 | the area around Bulunkul Lake | 37.706054,  72.973343 | *Leptolyngbya* sp. | *Nodularia spumigena* |
| F02 | 2017 | the area around Bulunkul Lake | 37.706375,  72.975134 | *Leptolyngbya* sp., *Phormidium* sp. | *Synechocystis* sp. PCC 7509, *Microcoleus acremanii* UTCC 313 |
| F03 | 2017 | the area around Sassykkul Lake | 37.678660,  73.183172 | *Leptolyngbya* sp., *Phormidium* sp., *Nodularia* sp. | *Synechococcus* sp. PCC 7336, *Leptolyngbya* sp. UIC 10125, *Leptolyngbya* sp. PCC 6406 |
| F04 | 2017 | the area around Sassykkul Lake | 37.67187,  73.133987 | *Leptolyngbya* sp. | *Nodularia spumigena* |
| F05 | 2017 | the area around Sassykkul Lake | 37.710374,  73.216816 | *Leptolyngbya* sp., *Phormidium* sp. | *Synechocystis* sp. PCC 7509, *Microcoleus acremanii* UTCC 313 |
| F06 | 2017 | the area around Khargush Lake | 37.446674,  73.072492 | *Leptolyngbya* sp., *Phormidium* sp. | *Microcoleus acremanii* UTCC 313 |
| F07 | 2017 | the area around Khargush Lake | 37.446025,  73.072038 | *Leptolyngbya* sp. | *Microcoleus acremanii* UTCC 313 |
| F08 | 2017 | the area around Khargush Lake area, mountain side | 37.444866,  73.065387 | *Leptolyngbya* sp., *Phormidium* sp. | *Rivularia* sp. VP4 08, *Microcoleus acremanii* UTCC 313, *Calothrix* sp. KVSF5, *Jaaginema geminatum* SAG 1459-8, *Leptolyngbya* sp. CYN68, *Leptolyngbya tenuis* PMC304.07 |
| F09 | 2017 | the area around Chukurkul Lake | 37.33136,  73.06609 | *Leptolyngbya* sp. | *Nodularia spumigena* |
| F10 | 2017 | the area around Rangkul Lake | 38.433559,  74.289177 | *Leptolyngbya* sp., *Gloeocapsopsis* sp. | *Synechocystis* sp. PCC 7509, *Pycnacronema arboriculum* 41PC, *Microcoleus acremanii* UTCC 313, *Leptolyngbya tenuis* PMC304.07 |
| F11 | 2017 | the area around Shorkul Lake | 38.26745,  74.10131 | *Leptolyngbya* sp. | no matches with like-weight ratio above 95% |
| F12 | 2017 | the area around Shorkul Lake area, mountain side | 38.27225,  74.11720 | *Leptolyngbya* sp. | *Nodularia spumigena* |
| F13 | 2017 | the area around Shorkul Lake area, mountain side | 38.24758,  74.11172 | *Leptolyngbya* sp. | *Geitlerinema* sp., *Leptolyngbya* sp. UIC 10125 |
| F14 | 2017 | the area around Karakul Lake | 39.040545,  73.576703 | *Leptolyngbya* sp., *Phormidium* sp., *Scytonema* sp., *Nostoc pruniforme* | *Synechococcus* sp., *S.* sp. PCC 7336, *Calothrix* sp. 96/26 LPP3, *Leptolyngbya* sp. PCC 6406 |
| F15 | 2017 | the area around Karakul Lake | 39.046579,  73.586434 | *Leptolyngbya* sp., *Phormidium* sp., *Calothrix* sp., *Chroococcus* sp., *Nostoc* sp. | *Capilliphycus* sp., *Synechococcus* sp., *Calothrix* sp. 96/26 LPP3 |
| F16 | 2017 | the area around Karakul Lake area | 39.058997,  73.592976 | no observed Oxyphotobacteria | *Gloeocapsa* sp., *Leptolyngbya* sp. UIC 10125, *Jaaginema geminatum* SAG 1459-8, *Leptolyngbya* sp. PCC 6406 |
| F17 | 2017 | the area around Karakul Lake road to the glacier | 39.278792,  73.303104 | *Leptolyngbya* sp., *Phormidium* sp. | *Rivularia* sp. VP4 08, *Nodularia spumigena, Microcoleus acremanii UTCC 313* |
| F18 | 2017 | the area around Karakul Lake, glacier foreground | 39.325893,  73.197212 | *Leptolyngbya* sp. | *Leptolyngbya* sp. UIC 10125 |
| F19 | 2017 | the area around Karakul Lake | 39.325893,  73.197280 | *Leptolyngbya* sp. | *Geitlerinema* sp., *Nodularia spumigena, Leptolyngbya sp. UIC 10125, Calothrix sp. 96/26 LPP3* |
| F20 | 2017 | the area around Karakul Lake | 39.3259086,  73.2031097 | *Leptolyngbya* sp., *Phormidium* sp., *Calothrix* sp., *Geitlerinema* sp. | *Microcoleus acremanii* UTCC 313 |
| F21 | 2017 | the area around Karakul Lake | 39.294777,  73.278610 | *Phormidium* sp. | *Rivularia* sp. VP4 08, *Nodularia spumigena, Calothrix sp. 96/26 LPP3* |
| F22 | 2017 | the area around Karakul Lake mountain side | 39.131588,  73.438736 | *Leptolyngbya* sp., *Phormidium* sp. | *Microcoleus acremanii* UTCC 313 |
| F23 | 2017 | the area around Karakul Lake | 39.131168,  73.438980 | no observed Oxyphotobacteria | *Synechococcus* sp. PCC 7336, *Synechocystis* sp. PCC 7509, *Jaaginema* sp. IkpSMP32, *Aerosakkonema funiforme* Lao35, *Microcoleus acremanii* UTCC 313 |
| F24 | 2017 | the area around Karakul Lake, mountain side | 39.142657,  73.435051 | no observed Oxyphotobacteria | *Nodularia spumigena* |
| F25 | 2017 | the area around Karakul Lake, mountain side | 39.143755,  73.426084 | *Leptolyngbya* sp., *Phormidium* sp., *Oscillatoria* sp. | *Nodularia spumigena*, *Calothrix* sp. 96/26 LPP3 |

Fig. S1. Comparison of the chemical properties of soil samples from California and Pamir, including electrical conductivity (EC [µS/cm]), pH, iron (Fe [mg/kg]), total nitrogen (TN [%]), total carbon (TC [%]), total magnesium (TMg [cmol/kg]), total sodium (TNa [cmol/kg]), total potassium (TK [cmol/kg]), and total calcium (TCa [cmol/kg]). Parameters with a statistically significant difference between the two environments (tested with a Kruskal-Wallis test) are marked with a red asterisk.


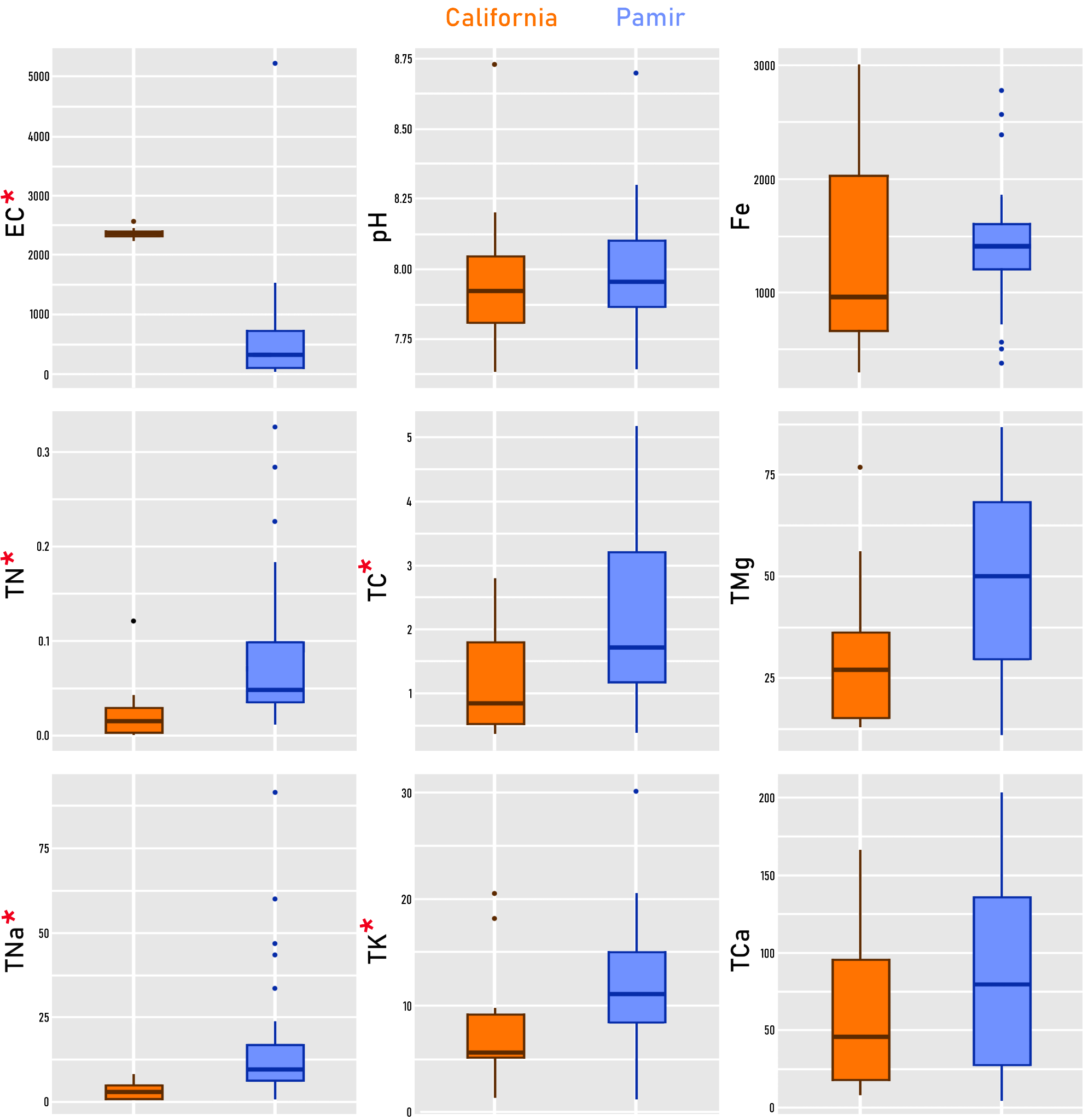

Supplement: Supplementary file 1 [file Data_Sheet_1.docx]
